# In Vivo Radiobiological Investigations with the TOP-IMPLART Proton Beam on a Medulloblastoma Mouse Model

**DOI:** 10.3390/ijms24098281

**Published:** 2023-05-05

**Authors:** Daniela Giovannini, Cinzia De Angelis, Maria Denise Astorino, Emiliano Fratini, Evaristo Cisbani, Giulia Bazzano, Alessandro Ampollini, Massimo Piccinini, Enrico Nichelatti, Emiliano Trinca, Paolo Nenzi, Mariateresa Mancuso, Luigi Picardi, Carmela Marino, Concetta Ronsivalle, Simonetta Pazzaglia

**Affiliations:** 1 ENEA SSPT-TECS-TEB, Casaccia Research Center, Division of Health Protection Technology (TECS), Agenzia Nazionale per le Nuove Tecnologie, l’Energia e lo Sviluppo Economico Sostenibile (ENEA), Via Anguillarese 301, 00123 Rome, Italy; daniela.giovannini@enea.it (D.G.); emiliano.fratini@enea.it (E.F.); mariateresa.mancuso@enea.it (M.M.); carmela.marino@enea.it (C.M.); 2 Istituto Superiore di Sanità (ISS), Viale Regina Elena 299, 00161 Rome, Italy; cinzia.deangelis@iss.it (C.D.A.); evaristo.cisbani@iss.it (E.C.); 3 ENEA FSN-TECFIS-APAM, Frascati Research Center, Via Enrico Fermi 45, 00044 Frascati, Italy; mariadenise.astorino@enea.it (M.D.A.); giulia.bazzano@enea.it (G.B.); alessandro.ampollini@enea.it (A.A.); emiliano.trinca@enea.it (E.T.); paolo.nenzi@enea.it (P.N.); luigi.picardi@enea.it (L.P.); concetta.ronsivalle@enea.it (C.R.); 4 ENEA FSN-TECFIS-MNF, Frascati Research Center, Via Enrico Fermi 45, 00044 Frascati, Italy; massimo.piccinini@enea.it; 5 ENEA FSN-TECFIS-MNF, Casaccia Research Center, Via Anguillarese 301, 00123 Rome, Italy; enrico.nichelatti@enea.it

**Keywords:** proton therapy, linac, dosimetry, small-field irradiation, preclinical mouse models, medulloblastoma, cerebellum, apoptosis

## Abstract

Protons are now increasingly used to treat pediatric medulloblastoma (MB) patients. We designed and characterized a setup to deliver proton beams for in vivo radiobiology experiments at a TOP-IMPLART facility, a prototype of a proton-therapy linear accelerator developed at the ENEA Frascati Research Center, with the goal of assessing the feasibility of TOP-IMPLART for small animal proton therapy research. Mice bearing Sonic-Hedgehog (Shh)-dependent MB in the flank were irradiated with protons to test whether irradiation could be restricted to a specific depth in the tumor tissue and to compare apoptosis induced by the same dose of protons or photons. In addition, the brains of neonatal mice at postnatal day 5 (P5), representing a very small target, were irradiated with 6 Gy of protons with two different collimated Spread-Out Bragg Peaks (SOBPs). Apoptosis was visualized by immunohistochemistry for the apoptotic marker caspase-3-activated, and quantified by Western blot. Our findings proved that protons could be delivered to the upper part while sparing the deepest part of MB. In addition, a comparison of the effectiveness of protons and photons revealed a very similar increase in the expression of cleaved caspase-3. Finally, by using a very small target, the brain of P5-neonatal mice, we demonstrated that the proton irradiation field reached the desired depth in brain tissue. Using the TOP-IMPLART accelerator we established setup and procedures for proton irradiation, suitable for translational preclinical studies. This is the first example of in vivo experiments performed with a “full-linac” proton-therapy accelerator.

## 1. Introduction

Medulloblastoma (MB) is the most common malignant brain tumor in children. The European annual incidence rate is 6.5 per million children [1]. There are several types of MB, which are distinguished based on histological classification or on genetic alterations. MB, genetically defined, is classified into WNT-activated, SHH-activated and *TP53*-mutant, SHH-activated and *TP53*-wild-type, and non-WNT/non-SHH groups [2]. In particular, about one-third of all MBs show aberrant activation of the SHH signaling pathway, a developmental axis in which the tumor suppressor gene *PATCHED1* (*PTCH1)* normally imposes an inhibitory effect [3]. Currently, multimodal treatment—surgery, radiotherapy, and chemotherapy—is the most effective strategy against MB. The conventional doses of RT delivered to the craniospinal axis and to the posterior fossa are 54–56 Gy. Using such doses, a high proportion of MB survivors have significant long-term consequences, including marked losses of Intelligence Quotient and endocrine dysfunction [4]. Many attempts have been made to control tumor growth while trying to reduce the radiation-related long-term neurocognitive effects, especially in young children. Recent strategies in pediatric oncology have also included the use of 3D conformal radiotherapy, as well as proton therapy, to minimize late effects. Protons are now increasingly used to treat pediatric MB patients, with important and clinically relevant differences compared to photon radiation treatments [5,6].

TOP-IMPLART (Intensity Modulated Proton Therapy Linear Accelerator for Radiotherapy) [7] is a pulsed proton linear accelerator (linac) for proton therapy applications developed at the ENEA Frascati Research Center. The machine, which consists of a sequence of accelerating modules, each increasing the maximum proton energy in steps of a few MeVs, is built in the framework of a national project funded by Lazio Innova-Regione Lazio and led by ENEA in collaboration with the Italian National Institute of Health (ISS) and Regina Elena Hospital [8,9]. The modularity of this type of accelerator allows extraction of the proton beam at the end of the last installed module for application in the bio-medical field and in particular for “in vitro” and “in vivo” radiobiology experiments. The output energy can be decreased from the maximum value, combining active and passive energy degradation techniques, to satisfy the requirements of different experiments in terms of penetration depth in the target volume.

The stability, reproducibility, and quality of the beam have been validated by radiobiological experiments combined with dosimetric characterization [10]. The entire process of characterization of TOP-IMPLART, supported by the results obtained with radiobiological in vitro experiments of cell killing and clonogenic survival experiments conducted with V79 and CHO cells (unpublished observation), allowed us to address the efforts toward an in vivo radiobiological experiment campaign in order to verify the suitability/potentialities of this facility for these kinds of studies. In fact, despite the steady worldwide increase in the number of proton therapy centers, proton radiobiology still has many open questions requiring either basic or translational preclinical research. Small animal proton therapy research may contribute to the basic understanding of in vivo radiation effects, but systems for proton radiotherapy research in these models are still rather limited, so far [11]. For proton research on small animal models, for instance, the position of the target volume and dosimetry are critical factors demanding specific solutions. Generally, murine models offer many advantages for mechanistic radiobiological investigations on normal tissue and tumor response, including short lifespan and the availability of genetically engineered mice to study the relevance of specific genes on radiation responses.

In this study, the proton therapy linear accelerator TOP-IMPLART was employed for in vivo radiobiological investigations on the effects of proton irradiation on a Shh-dependent MB mouse model, in both tumor and normal brain tissues. Proton beam parameters were successfully adapted to preclinical studies on mice as shown by the homogeneity of the dose distribution. Apoptotic response in MB tumor allografts after irradiation with protons or photons was compared to gain information on their biological effectiveness. In addition, by combining active and passive energy degradation techniques, we had reproducibly restricted the depth at which the proton radiation dose is delivered, carrying out the exposure of neonatal mouse brain at postnatal days 5 (P5) with two different collimated Spread-Out Bragg Peaks (SOBP) of 3 mm and 8 mm in depth. These results support TOP-IMPLART as an in vivo radiotherapy facility for in vivo proton beam research on mouse models, besides its use in proton therapy. This has implications for the open mechanistic research questions on proton radiobiology that limit the advancement in proton therapy research.

## 2. Results

Using a 55.5 MeV proton beam we created and delivered four different SOBPs. This was achieved by conveniently coupling a single range modulator (RM) with a range shifter (RS) of specific thickness (1.5 mm and 12.9 mm for tumors and 11.2 mm and 15.2 mm for neonatal mouse brain). In the following sections, the results of the proton beam characterization on target, in terms of energy distribution, lateral uniformity, and delivered dose reproducibility are reported. Details on the setup and the different components are discussed in Materials and Methods

### 2.1. Energy Characterization: Pristine and SOBP Measurement

**Figure 1:**
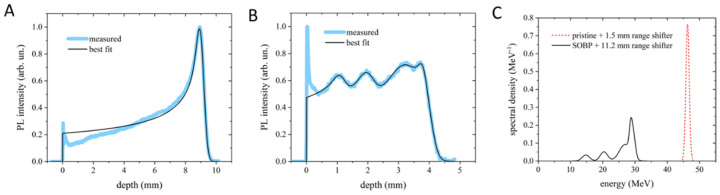
Bragg curve profiles of the PBP (**A**) and SOBP (**B**) obtained from the PL images stored in LiF crystals and their best-fitting curves. (**C**) Energy spectra corresponding to the best-fitting curves shown in (**A**,**B**). In (**A**,**B**), the peaks at zero depth are ascribed to light scattering at the crystal edge and were therefore excluded from the fitting process.

The dose distribution and the corresponding energy content were measured by the PL (PhotoLuminescence intensity profile vs. penetration depth) images stored in lithium fluoride (LiF) irradiated crystals. In Figure 1A,B, two representative results are provided for pristine Bragg peak (PBP) with RS of 1.5 mm and for SOBP with RS of 11.2 mm. For both cases, the experimental profile was best fitted using the analytical model described in [12]. The PBP allows us to estimate a peak energy value of 46.28 ± 0.32 MeV and an energy spread (standard deviation of the distribution) of 520 ± 30 keV under the assumption of a Gaussian energy distribution (Figure 1A). The SOBP (Figure 1B) shows five Gaussian components, whose central energies and spreads are reported in Table 1. In addition, the energy spectra resulting from the fitting process for the PBP and the SOBP are shown in Figure 1C. The ripple in the SOBP profile is due to the limited number of sectors in the custom-made RM.

### 2.2. Beam Stability, Transverse Profiles, and Dose Measurements

At the beginning of each irradiation session, the integral ionization chamber (iIC), which measures the absorbed dose to water in terms of monitor units and drives the proton irradiations, was calibrated relative to the microDiamond (mD) dose. The calibration was carried out for each experimental configuration with the mD placed at the target (mice) position (at 176.5 cm away from the beam pipe exit). A coefficient of variation (CV), defined as the ratio of the standard deviation of the measurements to the mean value, of CV = 8.5% was obtained for the 10 repeated measurements in the calibration conditions. This finding provides an estimation of the stability of the beam.

The mD signal is further cross-referenced using a real-time 2D ionization chamber (2D-IC) placed on the beam axis, 150 cm away from the beam pipe exit. The ratio of the 2D-IC signal to the mD dose was closely monitored and found to vary by no more than 2%. This ensures consistent beam stability monitoring during mouse irradiations when the mD is replaced with the target (mice). This intercalibration, between mD and 2D-IC, was also used for dose assessment: the combined uncertainty in 2D-IC dose values was estimated to be about 2.5%, obtained adding in quadrature the 1.5% uncertainty in the mD calibration and the above 2% statistical variability of the 2D-IC signal compared to the mD during the whole irradiation period. The reproducibility of the dose delivered in the several irradiation runs to the target, evaluated with the 2D-IC readings, was 1.6% for MB tumors and better than 7.6% for neonatal mouse brains. These results are consistent with the estimated beam stability.

**Figure 2:**
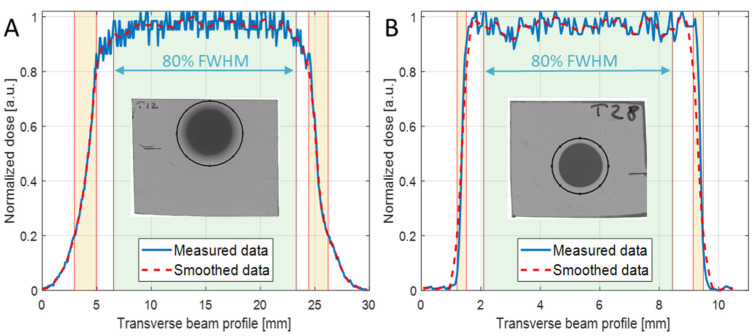
*X*-axis normalized dose profiles of the irradiated EBT3 films (solid blue line) and the relevant smoothed data (dashed red line) of (**A**) mouse tumor with 20 mm collimator diameter and SOBP = 19 mm, and (**B**) neonatal mouse brain with 8 mm collimator diameter and SOBP = 3 mm. The insets show the selected ROIs used to extract the curves.

For the irradiation of each mouse, a small piece of EBT3 Gafchromic film was placed transverse to the beam axis, in front of the mouse, to control the uniformity of the irradiation at the target surface and the target centering. Figure 2A,B report two representative examples of transverse (*x*-axis) beam profiles measured with EBT3 in MB tumor and neonatal mouse brain irradiations, respectively. The uniformity of the irradiation was evaluated, according to ICRU 78 and AAPM Task Group 224 Report, in terms of flatness and symmetry of the broad beam, in a region about the center of the beam profile corresponding to 80% of the field size of the normalized smoothed transverse dose profile (Figure 2A,B). In addition, penumbra parameters were also evaluated from EBT3 beam profiles in x and y directions, and are reported in Table 2 and Table 3 for the two collimators used. An additional check of the delivered dose to the target was also offered by the EBT3 film analysis; This information was not available during the irradiation session (real-time), rather, according to the adopted protocol for the detector calibration, it was available a few days after the irradiation session. The dose evaluated with 2D-IC and with EBT3 films agreed within 1%.

### 2.3. Apoptosis in MB Allografts after Proton Irradiation

**Figure 3:**
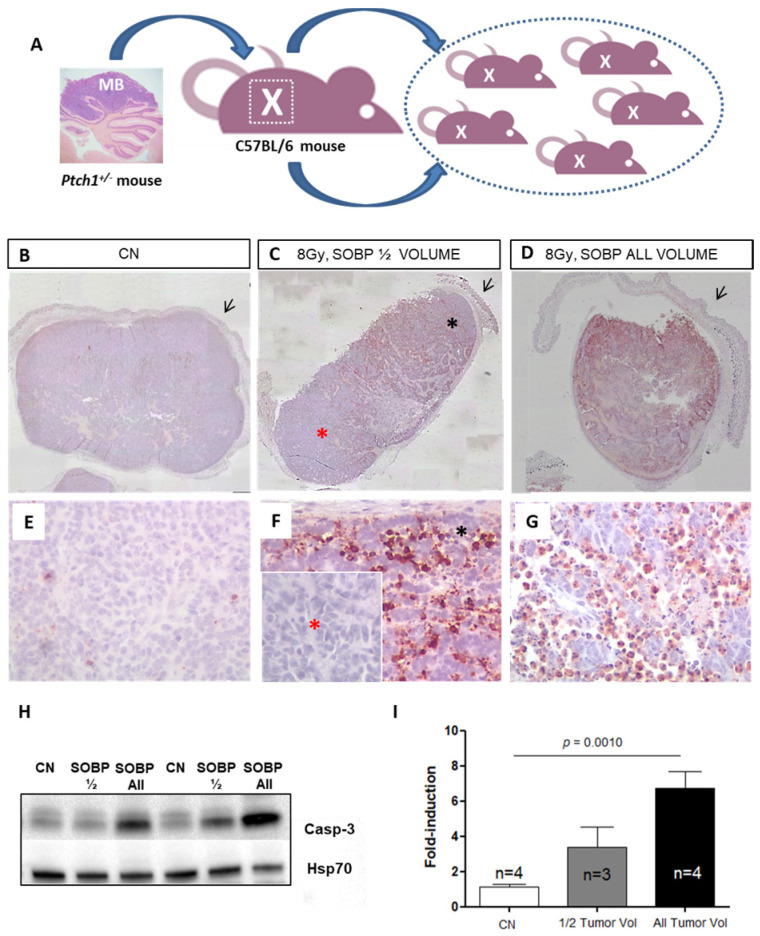
Schematic representation of the employed MB tumor model. Fragments of *Ptch1^+/^*^−^-derived tumor were subcutaneously propagated in the right flank of C57Bl6/J mice (**A**). Once the tumors reached 1400–2400 mm^3^, mice were irradiated or sham-exposed. Immunostaining for cleaved-caspase-3 in MBs 4 h after proton irradiation with 8 Gy ((**B**–**D**); magnification 4×). Caspase-3 staining shows that with a SOBP of 7 mm dose delivery can be successfully restricted to the upper half of the tumor (**C**) or administered to the whole tumor mass when a 19 mm SOBP was used (**D**); unirradiated control (**B**); black arrows indicate mouse skin. High power views of caspase-3 staining ((**E**–**G**); magnification 40×). * Black asterisk in (**F**) represents the exposed upper tumor part. * Red asterisk in (**F**) shows a lack of caspase-3 immunoreactivity, indicating tissue sparing from irradiation. (**H**) Representative immunoblotting for caspase-3 in tumors irradiated entirely or for half of the volume and relative representation of densitometric immunoblot analysis (**I**). The number of mice used for Western blot analyses is indicated in the graphs (n), Student’s *t*-test was used for the statistical analysis.

*Ptch1^+/−^* heterozygous knockout mice are a model of tumor predisposition. Among other tumor types, they develop MB with a spontaneous incidence of about 40% when the *Ptch1* deletion is expressed in a C57Bl/6 background [13,14]. For the radiobiological characterization of irradiation with the proton beams, we have developed a mouse allograft model, in which a large fragment of a spontaneous primary MB, developed in a C57Bl/6/*Ptch1^+/−^* female, was excised and implanted into the flank of an immunocompetent WT C57Bl/6 female (Figure 3A). Upon tumor growth, the mouse was sacrificed, and the tumor mass was excised and cut into small fragments (about 25 mm^3^) that were implanted in WT C57Bl/6 females (n = 40). Noteworthy, to guarantee the tumor homogeneity required to compare the effects of proton or photon irradiation, all the tumors were propagated from a single MB. Once the tumors reached 1400–2400 mm^3^, mice were randomized to (i) sham, (ii) protons, and (iii) photons experimental groups.

As a first step, we tested whether proton irradiation with 8 Gy could be restricted to a specific depth in the tumor tissue by using two SOBPs of 7 mm or 19 mm to irradiate half (upper) and the whole tumor mass (Figure 3C,D,F,G). The upper tumor orientation is determined by the presence of the skin (black arrow) on the histological tumor sections. In Figure 3C, by using immunohistochemistry (IHC) at 4 h post-irradiation, we showed that with a SOBP of 7 mm, we were able to deliver the dose to the upper tumor part, as indicated by the labeling for the apoptotic marker cleaved-caspase-3, while nearly no radiation dose was delivered to the deepest tumor part, as shown by the absence of caspase-3 labeling (Figure 3C,F). Instead, when a SOBP of 19 mm was employed, the caspase-3 staining at 4 h post-irradiation was homogeneous through the entire tumor mass (Figure 3D,G). Accordingly, Western blot analysis showed that a significant 4.64-fold increase (*p* = 0.001) in the expression of cleaved caspase-3 was induced after irradiation of the whole tumor while irradiation of half of the tumor volume only produced a 2.33-fold increase, which was not significant compared to unirradiated tumors (Figure 3H,I).

### 2.4. Cell Death in MB Tumor Allografts after Irradiation with Proton or Photon 

**Figure 4:**
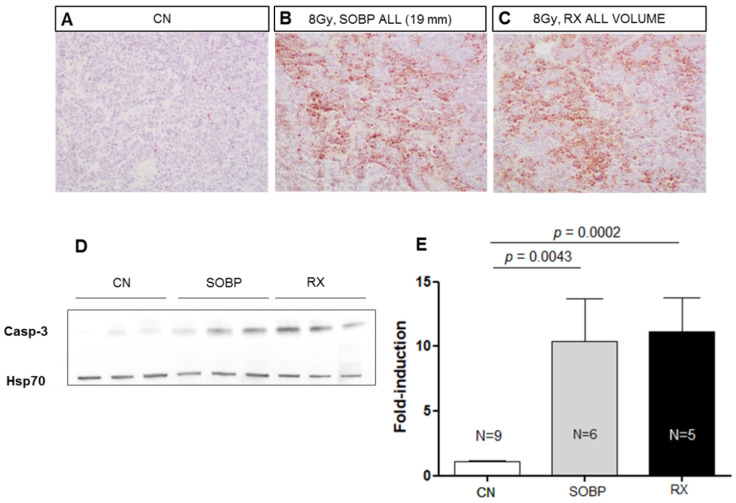
Representative immunostaining for cleaved-caspase-3 in MBs at 4 h after irradiation with 8 Gy of protons with a SOBP of 19 mm (46.4 MeV maximum energy) (**B**) or photons of 250 kVp (**C**). Unirradiated control (**A**). (**A**–**C**) are 20× magnification. Representative immunoblotting for caspase-3 in MBs at 4 h after irradiation in the same conditions reported above (**D**). (**E**) Graphic representation of densitometric immunoblot analysis in (**D**). The number of mice used for Western blot analyses is indicated in the graphs (n), Student’s *t*-test was used for the statistical analysis.

We next compared the effectiveness of 8 Gy of protons (46.4 MeV maximum energy) or photons (250 kVp) in inducing apoptosis in MB allografts at 4 h post-irradiation, using IHC and Western blot analyses (Figure 4A–E). The data showed that very similar significant increases of 9.51 and 10.25-fold in the expression of cleaved caspase-3 were induced after irradiation with 8 Gy of protons or photons compared to unirradiated tumors (*p* = 0.0043, and *p* = 0.0002, respectively) (Figure 4D,E). These findings suggested the same effectiveness of proton and photon irradiation in inducing apoptosis in MB in vivo.

### 2.5. Apoptosis Induced by Proton Irradiation on Neonatal Mouse Brain

**Figure 5:**
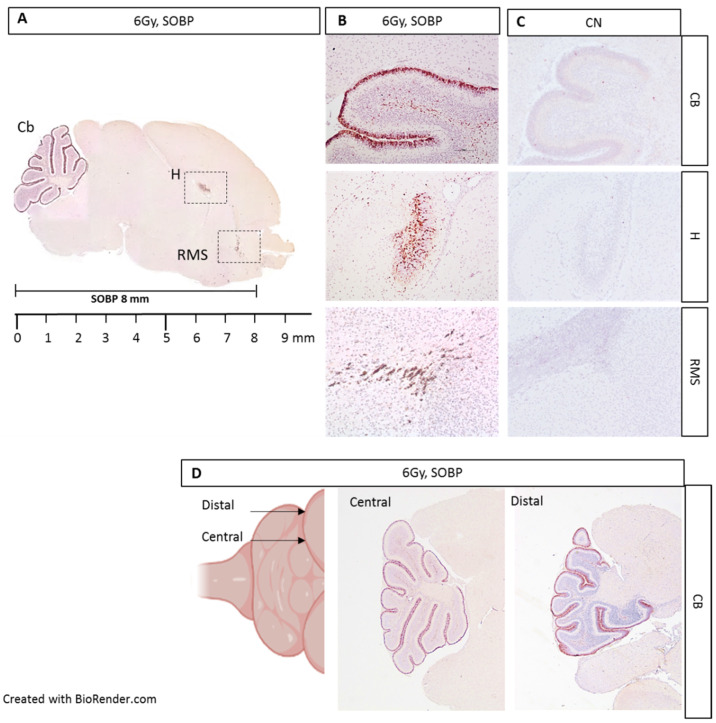
Immunostaining for cleaved-caspase-3 in brain sections from P5 mouse 4 h post-irradiation with 6 Gy of protons with a SOBP of 8 mm (29.5 MeV maximum energy) (**A**). Caspase-3 staining was especially detected in the EGL of the cerebellum (Cb), in the hippocampus (H), and in the rostral migratory stream (RMS) (**B**), compared to unexposed control (**C**). Serial cutting of sagittal brain sections showing the presence of caspase-3 labeling up to the distal end of the Cb (**D**). B and C are 10× and D is 4×.

We next moved to proton irradiation of neonatal mouse brain at postnatal day 5 (P5), representing a very small target, probing the adequacy and limitation of the positioning and dosimetry of the proton delivery system (Figure 5A–D). We first irradiated P5 mice with 6 Gy of protons with a SOBP of 8 mm and collected the brain at 4 h post-irradiation for IHC. Caspase-3 labeling was especially detected in the external granule layer (EGL) of the cerebellum, in the hippocampal region (H), and in the rostral migratory stream (RMS), representing regions of elevated radiosensitivity in the developing neonatal mouse brain (Figure 5A,B). The localization of caspase-3 staining was consistent with the SOBP width (8 mm) and the neonatal mouse brain size at P5 (about 9 mm). In addition, through the serial sagittal cutting of brain sections, we were able to detect caspase-3 labeling until the more distal lateral end of the cerebellum, indicating that the whole organ was within the irradiation field (Figure 5D).

**Figure 6:**
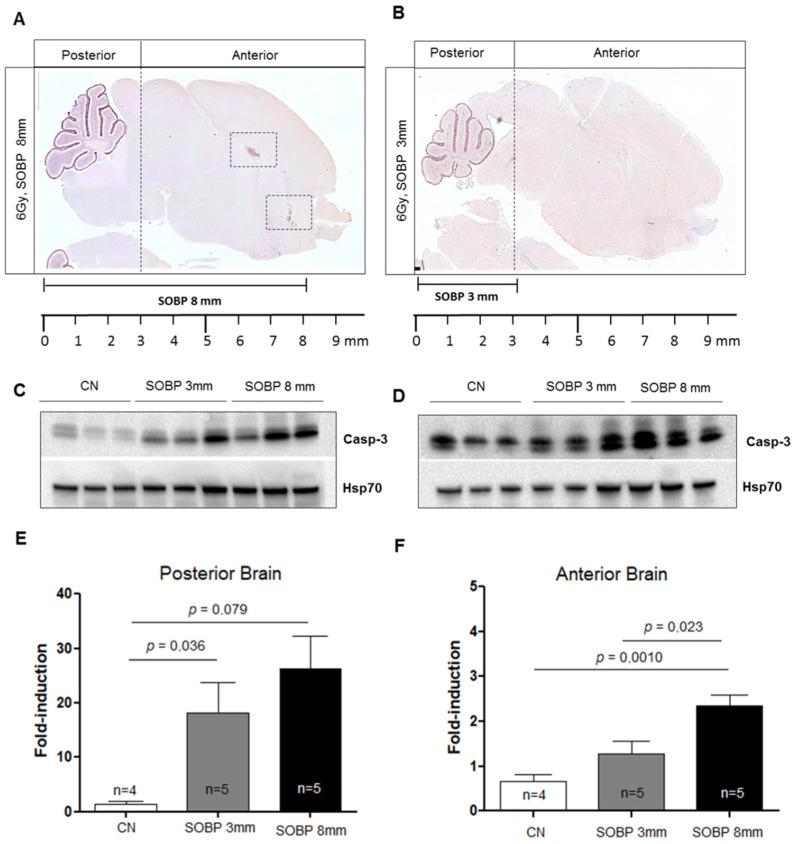
Immunostaining for cleaved-caspase-3 of brain sections at P5 after irradiation with 6 Gy of protons with SOBP of 3 mm (16.2 MeV maximum energy) (**A**) or 8 mm (29.5 MeV maximum energy) (**B**). Dashed vertical lines in A and B indicate the area of cut used to separate the posterior and anterior brain parts during the freezing collection. Dashed areas in A indicate H and RMS regions. Representative immunoblotting for caspase-3 in the posterior (**C**) or anterior brain part (**D**) and relative representation of densitometric immunoblot analyses (**E**,**F**). The number of mice used for Western blot analyses is indicated in the graphs (n), Student’s *t*-test was used for the statistical analysis.

We next evaluated the expression level of caspase-3 by IHC in P5 mouse brains irradiated with 6 Gy of protons with aSOBP of 8 mm or 3 mm at 4 h post-irradiation. Caspase-3 labeling was mainly localized in the EGL of the cerebellum for both SOBP of 8 mm and 3 mm (Figure 6A,B). However, while the H and RMS regions also show caspase-3 labeling with the 8 mm SOBP (Figure 6A), these areas were not labeled with the 3 mm SOBP (Figure 6B). Western blot analysis was also carried out, and the brain was cut coronally to separate the anterior and the posterior brain parts, which were analyzed separately. For the posterior brain portion, both irradiation with a SOBP of 8 mm and 3 mm significantly increased the expression of caspase-3, by 18.3-fold or 12.6-fold, respectively, compared to unirradiated control (*p* = 0.0079 and *p* = 0.036) (Figure 6C,E). Instead, for the anterior brain part, differences were observed between the SOBP of 8 mm and 3 mm (Figure 6D,F). A significant 3.5-fold increase (*p* = 0.001) in the expression of caspase-3 was only detected for irradiation with 8 mm SOBP. Instead, irradiation with 3 mm SOBP does not significantly increase the level of caspase-3 expression in the anterior brain, above those of unexposed control, indicating that this brain area was largely spared by radiation-induced tissue damage when the 3 mm SOBP was used.

## 3. Discussion

Preclinical in vivo studies are of paramount importance for translational research in radiation oncology. In particular, proton radiobiology still calls for basic and translational preclinical research [15]. Access to proton facilities and the availability of relevant in vivo animal models for proton research are both among the main bottlenecks limiting the advancements in the field of proton radiobiology and related clinical implementation to improve patient treatment.

Although access to proton facilities has progressively improved over the years, there is a paucity of published preclinical in vivo studies for protons, strongly soliciting the implementation of proton radiobiological facilities. In fact, radiobiological experiments mostly rely on clinical facilities, where the high beam energies used for patient treatments shall be degraded for the irradiation of small animals or even directly used in the plateau region of the pristine depth–dose curve [16,17,18,19,20]. Moreover, in clinical facilities, beam priority is correctly given to patient treatments.

The aim of this study was to investigate the feasibility of the proton beam generated by TOP-IMPLART, a prototype of a proton-therapy linear accelerator developed at ENEA Frascati Research Center, for irradiation of small targets represented by preclinical mouse models. Target sizes for in vivo radiobiological experimentation ranged from tumor mass of about 2 cm^3^ down to the size of the newborn mouse brain at P5 (9 mm). To the best of our knowledge, this is the first time that a proton-therapy system based on a full-linear accelerator has been employed for radiobiological in vivo experiments. For the experimental setup, a close interdisciplinary collaboration, involving engineers, physicists, and biologists, was required. The specific mode of proton energy deposition in penetrating materials, characterized by a maximum release (the Bragg peak) close to the end of the particles’ range, poses several critical issues that have to be addressed. In particular, the positioning of the target, indeed small positioning errors can cause irradiation of healthy tissue. Currently, at the TOP-IMPLART facility, positioning is performed manually, but it implies a very time-consuming procedure; therefore, improvements in the mouse housing are under development.

Another important aspect to be considered in the small animal irradiation is the homogeneity of the dose distribution; to this aim, the uniformity of the beam, in terms of flatness and symmetry, was investigated for both collimators used (20 mm and 8 mm in diameter). Results showed values of flatness <6% and symmetry <0.5% in the transverse profiles in x and y-directions for both collimators. These findings evidenced suitable characteristics of the TOP-IMPLART proton beam for small animal irradiation. Additionally, the agreement between the results obtained with the two collimators highlights that, on one side, a reduction in the collimator down to 8 mm in diameter does not affect the beam, and on the other side, a good alignment of the collimators in respect to the beam axis was achieved.

Several dosimetry systems, specifically dedicated to this proton beam, were designed and built inside this collaboration, for beam monitoring and dose assessment (2D-IC and iIC). These systems were complemented by commercial dosimetry systems such as mD and EBT3. Even if this approach provides redundant pieces of information, we consider it particularly relevant in the case of this accelerator (prototype of first proton linac characterized by high dose per pulse) used for in vivo experiments. It is extremely important to have immediate (real-time) information about the delivered dose or other irradiation parameters useful to define/check the good behavior of the system, which was achieved through the online detectors (2D-IC) developed within the TOP-IMPLART collaboration. Nevertheless, we consider an important step for the robustness of the results is the independent verification/checking of those quantities with more conventional dosimetry systems (i.e., Gafchromic films), suitable for these kinds of measurements. Unfortunately, data provided by films are not available during the irradiation session (real-time), but, as for all passive dosimetry systems, they can be provided after a period of time successive to the irradiation, which depends on the protocol used for the detector calibration.

From a biological point of view, the choice of the experimental model was made to reflect clinically relevant endpoints. In fact, we developed a model system involving subcutaneous transplantation into the mouse flank of a brain tumor developed in the *Ptch1* heterozygous mouse model [13,14]. This is relevant to clinics because SHH-dependent MB represents one-third of all MB cases [3], affecting mainly children less than 3 years of age, and because proton therapy for pediatric MB is currently used as an innovative approach with the potential to enhance the outcome of radiotherapy [21,22]. In addition, as an MB normal tissue counterpart, we employed the newborn mouse brain at P5, for its peculiar sensitivity to radiation effects at neonatal age [23]. On both these mouse model systems, to investigate tumor and normal tissue response, we employed an experimental approach to visualize the radiation-induced apoptosis through the assessment of the apoptotic marker caspase-3-activated by IHC. In addition, Western blot analysis was employed to quantify the apoptotic levels in tissue regions within and outside the proton irradiation field. The experimental data we produced unambiguously established that, by combining active and passive energy degradation techniques, we could reproducibly restrict the depth at which the proton radiation dose is delivered both in the tumor mass and in the newborn mouse brain, thus irradiating definite tissue volumes of organs and tumors, depending on the research question. Future investigations on the local control of proton therapy evaluating late local toxicities and pattern of tumor relapse in MB allografts after photon- or proton-irradiation are needed and clinically relevant.

Regarding the evaluation of the tumor response, xenografted tumor models are frequently used for in vivo proton relative biological effectiveness (RBE) estimation [24,25,26], while for normal tissue responses in vivo, the skin and the intestinal crypts are considered good model systems and are often employed to determine early and late tissue response to protons [27,28,29,30,31,32]. In particular, similar to our study, the endpoint of apoptosis was exploited to investigate the damage induced by protons in vivo in mouse intestinal tissue [33]. Further mouse experiments using the TOP-IMPLART accelerator and suitable mouse models might be foreseen to contribute to in vivo RBE calculation and to improve the mechanistic understanding of proton radiobiology.

The lack of dedicated small animal particle beam irradiators is recognized as a main limitation to advances in proton radiobiology and attempts are currently being made to develop experimental settings for preclinical in vivo studies, mostly based on adaptation of the technology for clinical particle beam irradiators [11,34,35]. Altogether, our findings demonstrated that the TOP-IMPLART accelerator and the related methodologies here developed, from the manual positioning of the target volume to the delivery of prescribed doses, through dosimetry and radiobiological endpoints, successfully fulfilled the requirements for a research platform for small animal studies.

## 4. Materials and Methods

### 4.1. Beam Line Design and Characteristics

**Figure 7:**
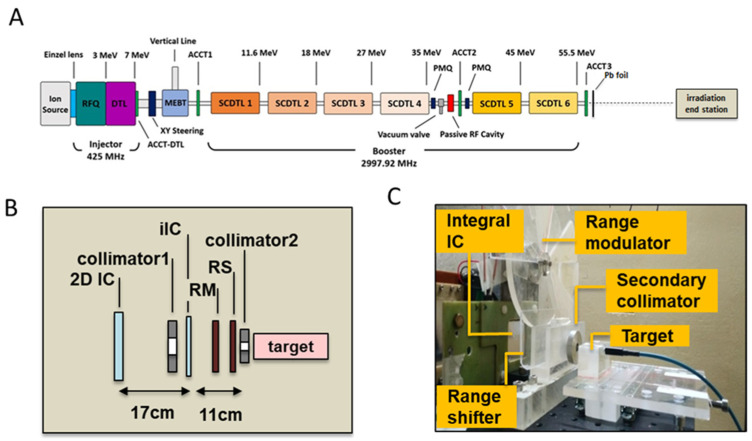
(**A**) Schematic layout of the TOP-IMPLART accelerator with maximum output energy of 55.5 MeV; Details of the irradiation end station set-up illustrating the schematic layout (**B**) and photograph (**C**); in picture (**C**) the target region hosts the mD dosimeter and its support for a dose assessment irradiation run.

The TOP-IMPLART accelerator is a radiofrequency (RF) pulsed fully linear machine consisting of a commercial injector operating at 425 MHz and an S-band section operating at 2997.92 MHz. The injector, consisting of a duoplasmatron source, an RFQ (Radio Frequency Quadrupole), a DTL (Drift Tube Linac), and their RF power source, accelerates the beam at 7 MeV. The S-band section consists of a sequence of Side-Coupled DTL (SCDTL) accelerating modules, powered by two 10 MW peak power klystrons. The measurements described in this paper were performed with the beam exiting from SCDTL-6 structure with a maximum energy of 55.5 MeV, as reflected by the schematic layout depicted in Figure 7A.

The proton beam propagates in vacuum in the accelerator and exits in air through a 50 µm titanium window. An AC current transformer (ACCT3) installed immediately after the vacuum window monitors the output current during the irradiation. Before the ACCT3, an aluminum foil of 1 mm of thickness is inserted to suppress the secondary electrons. The proton beam current used for this experiment was set to 10 µA in pulses of 2.4 µsec duration at a repetition frequency of 25 Hz. To obtain a homogeneous irradiation area at the target position, the beam is diffused first through a lead scattering foil (210 µm of thickness), placed in air at 18.5 mm after the vacuum window, then the protons move in air and reach the target position set at 176.5 mm from the linac exit. The irradiation end station, where the target is positioned (detailed in Figure 7B,C), is provided with online monitoring devices (ionization chambers), dosimetry diagnostics, collimators, a range shifter, and a custom-made range modulator, consisting of 7 plexiglass sectors of different thickness organized in a rotating wheel for generating the SOBP. Range shifters of different thicknesses were used to select different penetration depths.

The proton beam field was shaped through the use of aluminum collimators of different sizes. A first collimator (20 mm diameter) was employed in all the irradiation settings, and a second (8 mm diameter) was added to further reduce the beam size for irradiation of neonatal mouse brain. Irradiation was driven by an integral ionization chamber (iIC), placed close to the target position, immediately after the primary collimator. A second ionization chamber (2D-IC) acted as an independent dose monitor, calibrated by the mD.

**Figure 8:**
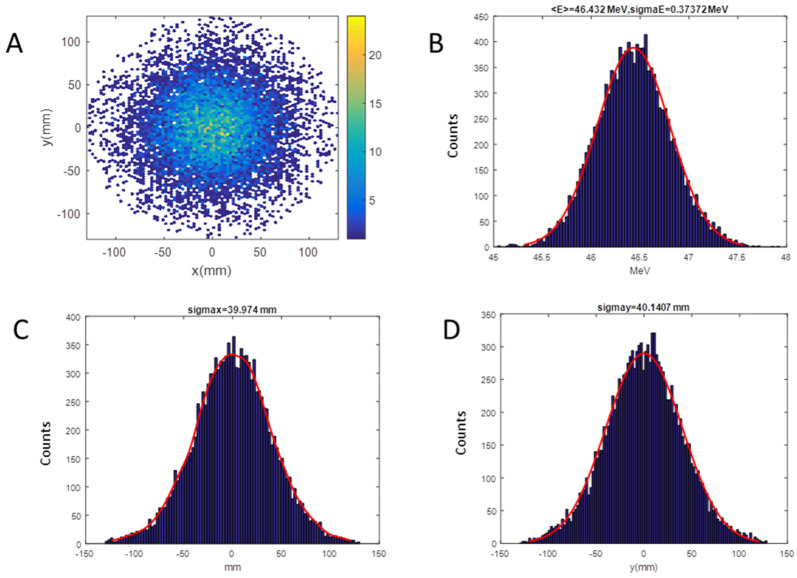
SRIM (Stopping and Range of Ions in Matter) output for the RS of 1.5 mm setup on target; Energy of 46.4 MeV, Energy spread of 373.7 keV: (**A**) Beam spot; (**B**) energy distribution; (**C**) horizontal distribution; (**D**) vertical distribution.

LINAC [36] and SRIM [37] codes were employed for the simulation of the beam dynamics inside the accelerator and the simulation of the irradiation line up to the target position, respectively: the beam coordinates describing positions, divergence, and energy of the beam before the titanium window, produced by the LINAC code, constitute the input data for SRIM to continue the propagation of the beam up to the target. As an example of this methodology, in Figure 8A,B, we show the computed beam spot at the target for the setup with a RS 1.5 mm thick. The final distribution has a Gaussian shape with a standard deviation of about 40 mm on x and y-axis (Figure 8C,D), corresponding to a homogeneity of 3% on the area defined by the collimator of 20 mm diameter, evaluated as the maximum variation. The pristine energy at the target surface, due to the interaction of protons with different materials, is decreased from 55.5 to 46.4 MeV.

### 4.2. Dosimetry

Several dosimetry systems were considered to experimentally characterize the TOP-IMPLART proton beam from a dosimetric point of view.

A diamond detector and two IC monitors provide the online control of the delivered dose, uniformity of transverse beam profile, and short and long-term stability of the beam. mD was also used for dose assessment and the preliminary calibration of the IC monitor. Offline EBT3 Gafchromic films and LiF crystals were used to complement/integrate/validate the data obtained with online systems, including the beam energy measurement.

The mD detector, model 60019 (PTW-Freinburg, Germany) connected to a Keithley electrometer (mod. 6517A with no polarizing voltage) was calibrated at the Italian Primary Standard Dosimetry Lab (ENEA-INMRI) in terms of absorbed dose to water in 60Co ɤ-rays.

The 2D-IC, a real-time 2D IC, developed within the TOP-IMPLART project, provides a pulse-by-pulse response. The 2D segmented IC in Micro Pattern Gaseous Detector technology is able to simultaneously acquire x/y strip readout with 0.3 mm spatial resolution, 100 fC sensitivity, and dynamic range greater than 10^4^. It is equipped with dedicated electronics that automatically adjust gain to the input collected charge through a feedback sample and hold capability.

**Figure 9:**
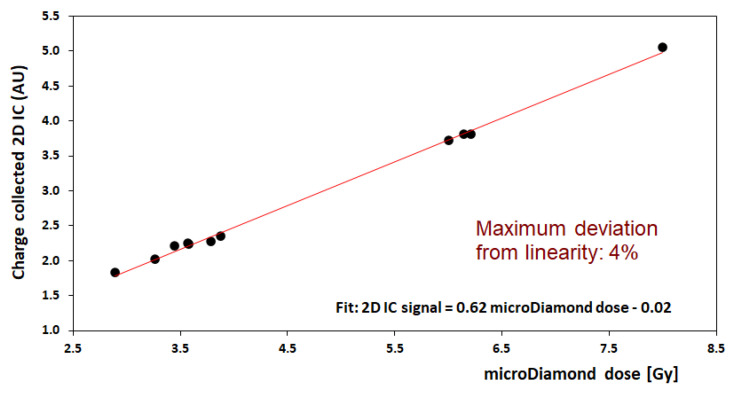
Calibration of dose measurement for the 2D-IC readings (in arbitrary unit, AU) against the microDiamond dosimeter (in Gy) for delivered doses in the range 2.7 to 8 Gy; this calibration corresponds to the 8 mm collimator configuration (neonatal brain irradiation).

A preliminary calibration of the 2D-IC was carried out using an mD detector. For this purpose, several irradiations at different dose values, in defined experimental configurations, were performed with the 2D-IC placed with its center on the beam axis at a fixed distance from the beamline exit and mD placed at the target position. The total charge values measured by the 2D-IC were recorded vs. mD dose; a good linearity response of the 2D-IC was verified in all configurations. An example of calibration performed using the 8 mm collimator is shown in Figure 9. The maximum deviation from linearity of the 2D-IC response was 4% in the (2.7–8) Gy dose range. This favorable behavior of the 2D-IC allows it to be used to monitor the stability of the output charge delivered by the accelerator during irradiation when the mD was removed and replaced by the actual target. In addition, 2D-IC provides accurate x and y profiles of the beam and its position, allowing, in such a way, the alignment of all systems (detectors, RS, RM, etc.) placed along the beamline to be checked; this piece of information is also relevant for a preliminary target positioning.

The iIC, an integral ionization chamber, is used to drive the irradiation. It operates at a bias voltage of 250 V (variable) and is realized with aluminized Mylar electrodes (12 μm Mylar, 4 μm aluminum) spaced by 2 mm of air. This chamber measures the dose in terms of monitor units and turns off the beam when a preset number of MUs (corresponding to the prescribed dose) is reached. The calibration of monitor units in terms of absorbed dose to water is performed by using the mD positioned in the place of the target (Figure 7C).

Gafchromic EBT3 (Ashland Advanced Materials) are self-developing dosimetry films with a symmetrical construction made of two 125 µm thick matte-polyester external layers and a 28 µm thick active middle layer. These radiochromic films have a dynamic dose range between 0.1 and 20 Gy, and a high spatial resolution, suitable to control beam uniformity. They have been digitized with an EPSON Expression 10,000XL/PRO flatbed color scanner. EBT3 films were calibrated at the Italian Primary Lab in ^60^Co source in terms of absorbed dose to water. In our experiment, they are specifically used during mice irradiations and placed in front of the target to evaluate the transverse beam profiles at the target positioning, and they also measure the absorbed dose of water delivered at the mouse surface.

Imaging exploiting the visible photoluminescence of color centers (CCs), created in LiF crystals by the proton beam, was employed to characterize the energy of the proton beam on the target and dose distribution with the depth of pristine Bragg peak and SOBP. Commercially available 15 × 15 mm^2^ LiF crystals with polished faces were irradiated with one of the 1 mm thick sides exposed perpendicularly to the impinging protons. For doses below a saturation threshold, the PL intensity is proportional to CCs concentration, which, in turn, increases linearly with the absorbed dose, and a luminescent image of the Bragg curve can be analyzed by means of a fluorescence microscope [38]. The high intrinsic spatial resolution of LiF crystals allows dose curve reconstruction with micrometric precision and determination of the energy beam parameters [39].

### 4.3. In Vivo Allograft and Study Design

Small fragments of murine MB tumors developed by a C57Bl/6/*Ptch1^+/^*^−^ female mouse [13,14] were transferred into the flank of immunocompetent WT C57Bl/6 females, and once the tumors reached 1400–2400 mm^3^, the mice were randomized to (i) sham, (ii) protons, and (iii) photons. Both proton and photon irradiations were single fractions.

### 4.4. Animal Irradiation

For tumor irradiation, tumor-bearing mice were anesthetized (65 mg/kg sodium pentobarbital i.p.), shielded by covering the whole mouse body, but for the tumor mass, with lead bars. Irradiation in photons was performed using a Gilardoni CHF 320G X-ray generator (Gilardoni S.p.A., Mandello del Lario, Italy; HVL = 1.6 mm Cu) operated at 250 kVp, 15 mA, with filters of 2.0 mm Al and 0.5 mm. For proton irradiation, the beam was delivered through a collimator of 20 mm in diameter to the flank tumors. Two different RS were used with thicknesses of 1.5 mm and 12.9 mm, respectively corresponding to maximum energies in water of 46.4 MeV (range in water 19.1 mm) and 25.8 MeV on target (range 6.6 mm). Additionally, the presence of the RM generated a different SOBP for each RS used with widths of 19 mm and 7 mm, respectively (Table 4, SRIM evaluations). The irradiation duration was around 95 s.

For neonatal irradiation, the proton dose was delivered to P5-neonatal mice restrained into an ad hoc plexiglass holder. SOBP widths of 3 and 8 mm were generated to deliver the dose through 8 mm diameter collimator at different tissue depths by using RS with thicknesses of 15.2 mm and 11.2 mm, corresponding to maximum energies in tissue of 16.2 MeV (range in water 2.9 mm) and 29.5 MeV (range in water 8.4 mm) (Table 5). It is worth noting that the amplitude of the SOBP coincides, within the uncertainty, with the range of the proton’s maximum energy, indicating that the entire thickness of the target is covered by the SOBP. The irradiation time was around 30 s.

### 4.5. Histological and Immunohistochemical Analysis

After irradiation, tumors and cerebella of C57Bl/6 pups were processed for histology by standard techniques, and tissue sections were cut (4 μm) for H&E staining and microscopic morphological examination. Fixed tissue sections were immunostained as described [40] using an antibody against cleaved-caspase-3 (Asp 175) (dilution 1:150 overnight; Cell Signaling Technology CS9664, Danvers, MA, USA). Samples were analyzed by light microscopy using the software NIS-Elements BR 4.00.05 (Nikon Instruments Europe B.V.; Florence, Italy).

### 4.6. Western Blot

Brain and tumor samples were collected at 4 h post-treatment, soaked in RNA later, and conserved at 5 °C. Proteins from tumors and cerebella at P5 were extracted, normalized, separated, and immunoblotted as described [41]. To evaluate apoptosis, cleaved-caspase-3 (Asp 175) rabbit polyclonal antibody was used (dilution 1:1000 overnight; Cell Signaling Technology, Danvers, MA, USA). Monoclonal antibody against HSP70 (dilution 1:10.000 30 min, Sigma-Aldrich H5147, St. Louis, MI, USA) was used as loading control. Specific proteins were visualized using ECL™ Prime Western Blotting Detection Reagent (Cytiva RPN2232) with ChemiDoc system XRS+ Biorad and quantified using ImageJ software version 1.8.0.

### 4.7. Statistical Analysis

Data were given as mean ± standard error of the mean (SEM). All statistical analyses were carried out using GraphPad Prism 6 statistical software (GraphPad, San Diego, CA, USA). The Kolmogorov–Smirnov test was used to verify that data were sampled from populations following the Gaussian distribution. Comparisons between groups were performed using the parametric t-test (significance taken as *p* < 0.05).

## 5. Conclusions

Combining the TOP-IMPLART proton-therapy accelerator with pre-clinically relevant mouse models provides an important preclinical setting to test a variety of questions on proton radiobiology on both tumor and normal tissues. Open mechanistic research questions on proton radiobiology—including normal tissue toxicity, differences in biological responses after proton and photon irradiation, and a reduction in uncertainties of the proton RBE at the end of the SOBP—might greatly benefit from the results of the research here presented. In fact, these results extend the experimental possibilities and pave the road for further mechanistic radiobiological investigations to compare in vivo the oncogenic effect of protons delivered by a pulsed fully linear accelerator vs. a comparable dose of photons in *Patched1* heterozygous (*Ptch1^+/^*^−^) mice, a mouse model of radiation hypersensitivity with a predisposition to cancer and non-cancer radiation-induced pathologies, including MB and lens opacity.

Despite the current limitations of the dose delivery system (manual positioning, custom range modulator, and range shifter), which are under improvement in different directions (laser-controlled positioning, implementation of magnetic proton beam scanning for a more efficient utilization of the beam), the TOP-IMPLART has demonstrated adequate performance for small animal in vivo studies by using a dose rate of about 0.2 Gy/sec for neonatal mice and 0.08 Gy/sec for MB mice.

However, the flexibility of the machine in terms of output pulse current and repetition frequency combined with the replacement of the actual passive beam delivery with an active modality could provide an increase in the dose rate of up to a factor of sixty. This will be exploited for the investigation of unconventional in vivo irradiation modalities.

## Figures and Tables

**Table 1:** SOBP energy component values and their spreads obtained from the best-fit process.

Energy (MeV)	Energy Spread (keV)	Relative Weight (%)
29.47 ± 0.77	639 ± 317	20.1
28.65 ± 0.31	457 ± 158	14.3
26.91 ± 0.26	1768 ± 126	41.1
20.35 ± 0.14	1142 ± 62	14.7
14.76 ± 0.11	1164 ± 78	9.8

**Table 2:** Flatness and symmetry of transverse profiles (in x- and y-directions) gained from EBT3 irradiation at the target surface, for the two collimators used.

	x Profile		y Profile	
Collimator	Flatness (%)	Symmetry (%)	Flatness (%)	Symmetry (%)
20 mm	4.2	0.24	5.6	0.32
8 mm	4.3	0.23	5.2	1.3

**Table 3:** FWHM and lateral penumbra of transverse profiles (x- and y-directions) gained from EBT3 irradiation at the target surface, for the two collimators used.

	x Profile	y Profile	x Profile	y Profile
Collimator	FWHM (mm)		Lateral Penumbra (mm)	
20 mm	20.94	21.01	2.02–1.79	2.04–1.72
8 mm	7.94	7.96	0.31–0.34	0.48–0.47

**Table 4:** Characteristics of proton beam in terms of specific range shifter used and corresponding maximum energy and range of the beam and SOBP width evaluated with SRIM code for MB irradiation.

RS (mm)	SOBP Width (mm)	Maximum Energy in Water (MeV)	Range in Water (mm)
1.5	19	46.4	19.1
12.9	7	25.8	6.6

**Table 5:** Characteristics of proton beam in terms of specific range shifter used and corresponding maximum energy and range of the beam and SOBP width evaluated with SRIM code for P5-neonatal mice irradiation.

RS (mm)	SOBP Width (mm)	Maximum Energy in Water (MeV)	Range in Water (mm)
11.2	8	29.5	8.4
15.2	3	16.2	2.9

## Data Availability

Other datasets analyzed during the study are available from the corresponding authors on reasonable request.

## Abbreviations

ACCT	AC current transformer
Cb	Cerebellum
CC	Color Centers
CV	Coefficient of variation
DTL	Drift Tube Linac
EGL	External Granule Layer
H	Hippocampus
Hsp70	Heat shock protein 70 kilodaltons
HVL	Half Value Layer
IHC	Immunohistochemistry
iIC	integral Ionization Chamber
IC	Ionization Chamber
LINAC	Linear accelerator
LiF	Lithium Fluoride
MB	Medulloblastoma
mD	microDiamonds
MU	Monitor Unit
PBP	Pristine Bragg Peak
P	Postnatal day
PL	Photo Luminescence
*Ptch1*	*Patched1*
RFQ	Radio Frequency Quadrupole
RM	Range Modulator
ROI	Region of Interest
RS	Range Shifter
SCDTL	Side Coupled DTL
SOBP	Spread Out Bragg Peaks
SRIM	Stopping and Range of Ions in Matter Intensity
TOP-IMPLART	Modulated Proton Therapy Linear Accelerator for Radiotherapy
WNT	Wingless/Integrated
WT	Wild Type
